# END-OF-LIFE DECISION SUPPORT NEEDS OF DEMENTIA FAMILY CAREGIVERS

**DOI:** 10.1093/geroni/igz038.2203

**Published:** 2019-11-08

**Authors:** Jung Kwak, Lisa Geshell

**Affiliations:** 1 The University of Texas at Austin, Austin, Texas, United States; 2 School of Nursing, The University of Texas at Austin, Austin, Texas, United States

## Abstract

One third of older adults die with dementia. At the end of life (EOL), persons with dementia require surrogate decision-makers, often their family caregivers, to make important EOL decisions. However, only a handful of evidence-based interventions exist to guide dementia caregivers in surrogate-decision making. In order to examine the acceptability and appropriateness of a decision coaching intervention developed for dementia caregivers, we conducted cognitive interviews (n=4), and one focus group (n=9) with dementia caregivers, and two focus groups with healthcare professionals (n=14) from a large healthcare system and a managed long-term care organization. Guiding questions for interviews and focus groups included: (1) types of decisions (what), and circumstances or triggers (when and how) that call for decision-making support by healthcare professionals, (2) barriers to families receiving decision-making support, and (3) decision support needs of family caregivers. All face-to-face interviews were audio-recorded, transcribed verbatim, and verified for accuracy. Content analysis was conducted to identify and organize themes and patterns emerging from the interview transcripts. Two main themes and subthemes emerged: (1) decision-making challenges and barriers: lack of advance care planning, caregivers’ acquiescence with dementia progression and caregiving role, discontinuing life sustaining therapies, and lack of communication between providers; and (2) decision support for families: advance care planning at different stages of dementia, preparing caregivers for life after the patient’s death, and providing adequate information about benefits and harms of treatment options specific to the practical concerns of patient and family caregivers. These findings provide implications for practice and future research.

